# Dehydroabietic Acid Suppresses Inflammatory Response Via Suppression of Src-, Syk-, and TAK1-Mediated Pathways

**DOI:** 10.3390/ijms20071593

**Published:** 2019-03-29

**Authors:** Eunji Kim, Young-Gyu Kang, Yong-Jin Kim, Tae Ryong Lee, Byong Chul Yoo, Minkyeong Jo, Ji Hye Kim, Jong-Hoon Kim, Donghyun Kim, Jae Youl Cho

**Affiliations:** 1 Department of Integrative Biotechnology, Sungkyunkwan University, Suwon 16419, Korea; im144069@gmail.com (E.K.); whalsrud1017@naver.com (M.J.); kjhmlkjhml@hanmail.net (J.H.K.); 2 Basic Research & Innovation Division, R&D Center, AmorePacific Corporation, Yongin 17074, Korea; kangyg82@amorepacific.com (Y.-G.K.); jaykim@amorepacific.com (Y.-J.K.); trlee@amorepacific.com (T.R.L.); 3 Colorectal Cancer Branch, Research Institute, National Cancer Center, Goyang 10408, Korea; yoo_akh@ncc.re.kr; 4 Department of Physiology, College of Veterinary Medicine, Chonbuk National University, Iksan 54596, Korea

**Keywords:** dehydroabietic acid (DAA), inflammation, NF-κB, AP-1

## Abstract

Dehydroabietic acid (DAA) is a naturally occurring diterpene resin acid derived from coniferous plants such as *Pinus* and *Picea*. Various bioactive effects of DAA have been studied including antibacterial, antifungal, and anticancer activities. However, the anti-inflammatory mechanism of DAA remains unclear. We evaluated the anti-inflammatory effect of DAA in macrophage cell lines. Dehydroabietic acid clearly reduced nitric oxide (NO) production and inflammatory gene expression decreased according to RT-PCR results. Dehydroabietic acid displayed anti-inflammatory activity at the transcriptional level in results from NF-κB- or AP-1-mediated luciferase assays. To identify the DAA target protein, we investigated NF-κB and AP-1 pathways by Western blotting analysis. Dehydroabietic acid suppressed the activity of proto-oncogene tyrosine protein kinase (Src) and spleen tyrosine kinase (Syk) in the NF-κB cascade and transforming growth factor beta-activated kinase 1 (TAK1) in the AP-1 cascade. Using overexpression strategies, we confirmed that DAA targeted these kinases. Our findings demonstrate the anti-inflammatory effects and molecular mechanism of DAA. This suggests that DAA has potential as a drug or supplement to ameliorate inflammation.

## 1. Introduction

Inflammation is an innate defense system of the mammalian body against pathogens. This first line of defense is activated to remove invading pathogens accompanying fever, swelling, pain, and redness [1,2,3]. Immune cells such as monocytes, macrophages, and neutrophils are rapidly recruited to inflamed sites, recognize foreign invaders, and release chemical mediators (cytokines, chemokines, and eicosanoids) [4,5]. In the process of recognition, pathogen recognition receptors (PRRs) of cells need to form a complex with the pathogens’ conserved structure called pathogen-associated molecular patterns (PAMPs) [2,6]. Toll-like receptors (TLRs) are one of the PRRs and are classified into 10 types. Each TLR detects different types of activators including lipids, lipoproteins, glycans, and nucleic acids and initiates the inflammatory signal activation [6,7]. The TLR adaptor molecules myeloid differentiation primary response 88 (MyD88) and TIR-domain-containing adaptor-inducing interferon-β (TRIF) transduce the signal to downstream molecules and finally activate inflammatory transcriptional factors such as nuclear factor (NF)-κB, activating protein (AP)-1, or interferon regulatory factors (IRFs) [6,8]. In NF-κB signaling, Src and Syk kinases are involved and transduce the activities to downstream molecules by phosphorylation [9]. Phosphorylated IκBα is degraded by ubiquitination, and segregated NF-κB translocates to the nucleus for inflammatory gene transcription. In the case of AP-1, activating signals from TLRs are delivered through the interleukin-1 receptor-associated kinases (IRAKs)/TAK1/MAPKs pathway [10,11,12]. Activated mitogen-activated protein kinases (MAPKs) phosphorylate AP-1 subunits including those of the Jun family (c-Jun, JunB, and JunD), Fos family (c-Fos, FosB, Fra-1, and Fra-2), and the activating transcription factor (ATF) family (ATF1, ATF2, and ATF3) for activation [13]. Both NF-κB and AP-1 play roles as transcriptional factors to produce inflammatory cytokines and chemokines (i.e., tumor necrosis factor (TNF)-α interleukin (IL)-1β, IL-6, cyclooxygenase (COX)-2, and inducible nitric oxide synthase (iNOS)) [14,15,16].

**Figure 1:**
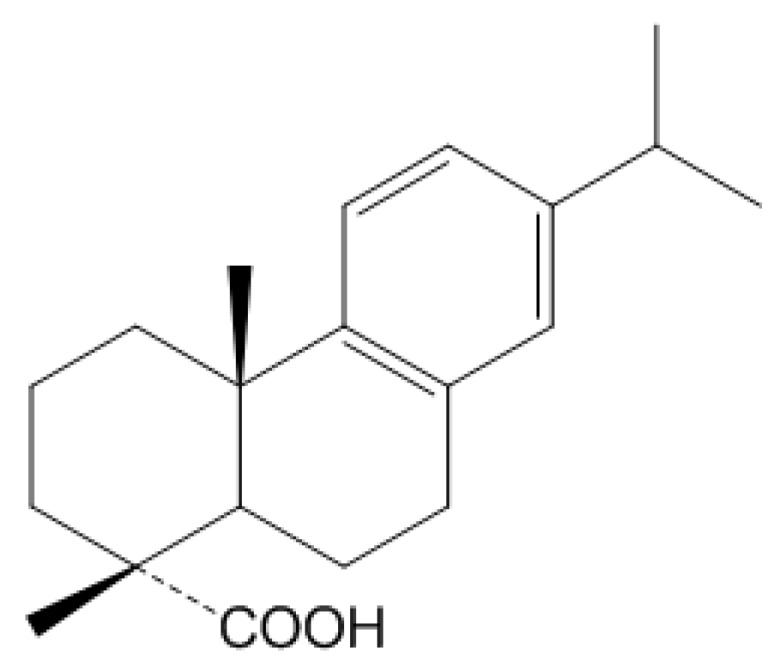
Structure of dehydroabietic acid (DAA).

Dehydroabietic acid (DAA) (Figure 1) is, along with abietic acid, a major compound of rosin derived from coniferous plants such as *Pinus*, *Picea*, *Larix*, and *Abies* [17,18,19]. Abietic acids are known to have biological activity including anti-inflammation or anti-allergy, and DAA has been studied as a peroxisome proliferator-activated receptor (PPAR) ligand in macrophages to suppress inflammation [19,20,21,22]. However, the detailed regulatory mechanism in inflammatory responses has not been deciphered. In this study, we confirmed that DAA reduced inflammatory mediators and gene expression. The mechanism by which DAA suppresses inflammatory response was investigated by luciferase assay and Western blotting analysis.

## 2. Results

### 2.1. The Effect of DAA on Nitric Oxide Production

**Figure 2:**
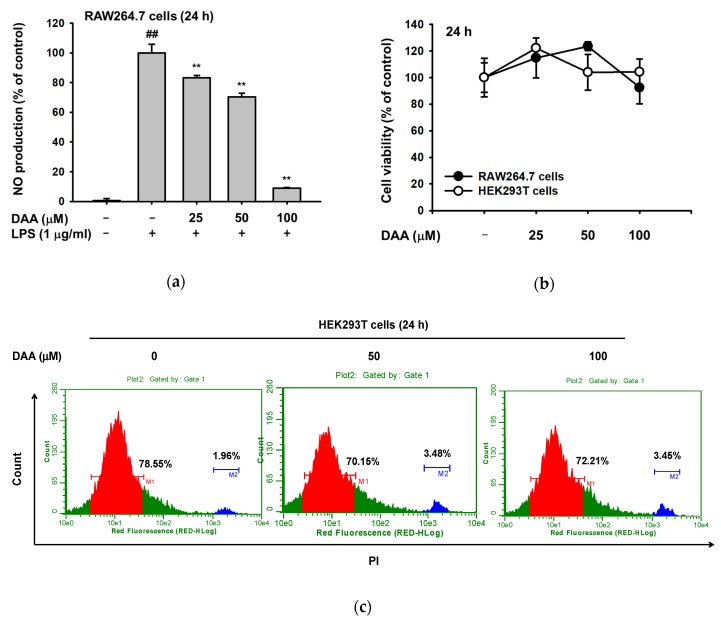
Effects of DAA on NO production and cytotoxicity. (**a**) DAA (0–100 µM) was pre-treated for 30 min, and LPS (1 µg/mL) was treated on RAW264.7 cells for 24 h. Cell supernatants were collected, and the production of NO was measured by Griess assay. (**b**) Cytotoxicity of DAA in RAW264.7 cells and HEK293T cells. Cells were incubated with DAA (0–100 µM) for 24 h, and then conventional MTT assay was performed. ^##^ *p* < 0.01 compared to the normal group, ** *p* < 0.01 compared to the induced group. (**c**) Cytotoxicity of DAA in HEK293T cells was examined by PI staining analysis. The percentage of cell death was analyzed by flow cytometry.

To examine whether DAA has an anti-inflammatory effect, we first investigated the production of nitric oxide (NO) under lipopolysaccharide (LPS) induction conditions in RAW264.7 cells. Dehydroabietic acid decreased NO production, with a significant reduction at 100 µM DAA (Figure 2a). Dehydroabietic acid was not toxic in both RAW264.7 and HEK293 cells, according to 3-[4,5-dimethylthiazole-2-yl]-2,5-diphenyltetrazolium bromide (MTT) assay and propidium iodide (PI) staining experiments (Figure 2b,c).

### 2.2. The Anti-Inflammatory Effect of DAA at the Transcriptional Level

**Figure 3:**
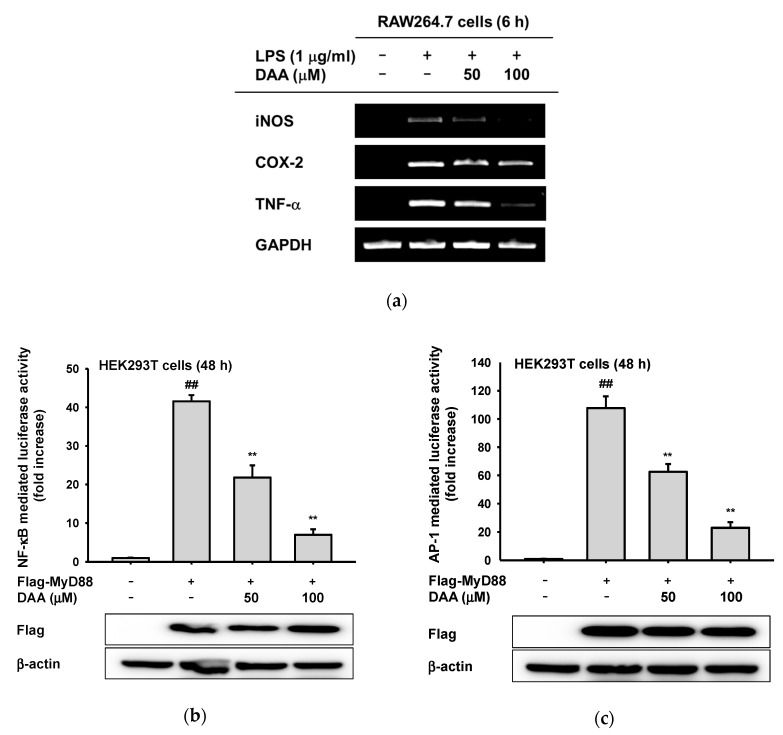
Effect of DAA on inflammatory transcriptional activation. (**a**) RAW264.7 cells were incubated with DAA (0–100 µM) in the presence or absence of LPS (1 µg/mL) for 6 h. Total mRNA was prepared from cells using Trizol methods, described in the Materials and Methods section. mRNA levels of inducible nitric oxide (iNOS), cyclooxygenase (COX)-2, and tumor necrosis factor (TNF)-α were determined by semiquantitative PCR. (**b**,**c**) Flag-MyD88 was transfected into HEK293T cells using polyethylenimine (PEI) with nuclear factor (NF)-κB-Luc or activator protein (AP)-1-Luc constructs, respectively. β-galactosidase plasmid was used as a control. DAA was treated for an additional 24 h, and luciferase activity was measured by a luminometer. The expression level of Flag-MyD88 (Lower panels of **b** and **c**) was examined by Western blotting. Antibodies against Flag and β-actin were used. Relative intensity (**b**,**c**) was values of the ratios calculated using densitometric scanning values of tagging protein (Flag) and densitometric scanning values of β-actin by the DNR Bio-imaging system (Gelquant software Version 2.7). ^##^ *p* < 0.01 compared to the normal group, ** *p* < 0.01 compared to the induced group.

Since DAA reduced NO production, we explored the regulatory mechanism of DAA in TLR4-mediated inflammatory responses. First, we conducted semiquantitative PCR to investigate how DAA modulates inflammatory reaction at the transcriptional level. The mRNA expression levels of inflammatory mediators including inducible nitric oxide (iNOS) and TNF-α were significantly reduced. Cyclooxygenase (COX)-2 level was also slightly affected by DAA (Figure 3a). Then, transcriptional factors influenced by DAA were analyzed by luciferase assay. We transfected TLR4 adaptor molecule myeloid differentiation primary response 88 (MyD88) to induce inflammatory signaling in HEK293T cells and determined NF-κB- or AP-1-mediated luciferase activity [8]. Dehydroabietic acid (0–100 µM) was non-toxic to HEK293T cells (Figure 2b,c), so DAA was treated on transfected HEK293T cells for 24 h. With DAA, MyD88-induced NF-κB and AP-1 transcriptional activities were meaningfully reduced at 100 µM (Figure 3b,c). Additionally, we confirmed the expression level of Flag-MyD88 by Western blotting to support these data.

### 2.3. The Anti-Inflammatory Effect of DAA on the NF-κB Signaling Pathway

**Figure 4:**
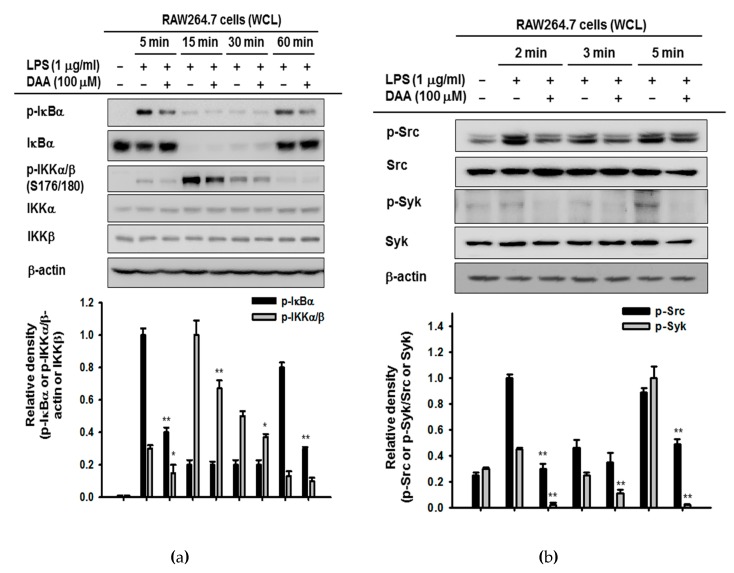

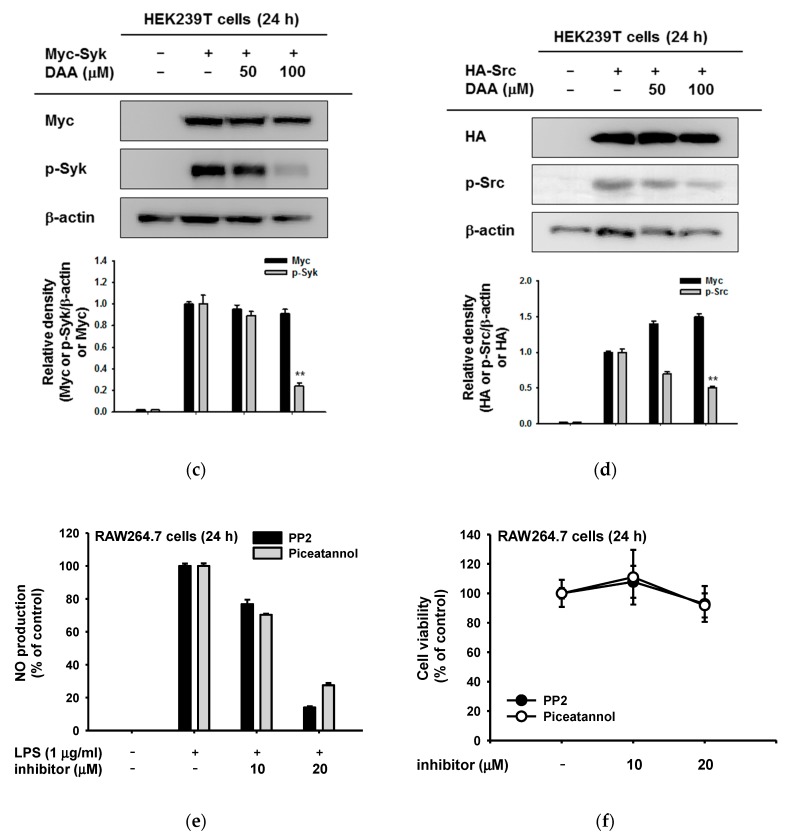
Effect of DAA in the NF-κB signaling cascade. (**a**,**b**) RAW264.7 cells were pre-treated with DAA, and LPS was applied in a time-dependent manner. Whole cell lysates were prepared, and immunoblotting was performed. Antibodies against phosphorylated or total IκBα, IKKα/β (serine 176/180), Src, Syk, and β-actin were used. (**c**,**d**) Myc-Syk or human influenza hemagglutinin (HA)-Src plasmids were transfected into HEK293T cells for 24 h, and then DAA (0–100 µM) was applied for an additional 24 h. Phosphorylated Syk and Src, Myc, HA, and β-actin were detected in whole cell lysates by immunoblotting. (**e**) (4-amino-5-(4-chlorophenyl)-7-(t-butyl) pyrazolo[3,4-d]pyrimidine) (PP2) or piceatannol (0–20 µM) was pre-treated on RAW264.7 cells for 30 min, and LPS (1 µg/mL) was treated for additional 24 h. NO production was determined by Griess assay. (**f**) Cytotoxicity of PP2 or piceatannol on RAW264.7 cells was examined by MTT assay. WCLs: whole cell lysates. Relative intensity (**b**,**c**) was the calculated ratio using densitometric scanning values of phospho-proteins and densitometric scanning values of β-actin or total forms of proteins from blots observed with independent repeats (*n* = 3) by the DNR Bio-imaging system (Gelquant software Version 2.7). * *p* < 0.05 and ** *p* < 0.01 compared to the induced group.

Based on the results of the luciferase assay, we screened the NF-κB and AP-1 signaling pathways to identify target proteins of DAA. By Western blotting analysis, phosphorylated signaling molecules were detected in a time-dependent manner. In the NF-κB pathway, phosphorylation of IκBα was blocked by DAA at 5 and 15 min, and phosphorylated IκB kinase (IKK)α/β (serine 176/180) was decreased at 5 and 15 min, without showing decreased levels of IKKα and IKKβ (Figure 4a). Since DAA regulated IκBα and IKKα/β at early time points (at 5 min), we prepared whole lysates of LPS-treated RAW264.7 cells in a brief time (2, 3, and 5 min) with DAA to assess activation of Src and Syk kinases. Src and Syk have been thought to be upstream molecules of NF-κB activation, and the blockade of Src and Syk at early time points affects the phosphorylated state of IκBα [23,24,25]. Activities of Src and Syk were suppressed by DAA at 3 min and at 3 and 5 min, respectively (Figure 4b). To clarify DAA targeted proteins, we determined activation of Src and Syk by an overexpression strategy. As correlated with previous results, DAA inactivated both Src and Syk kinases in Src- or Syk-overexpressing HEK293T cells (Figure 4c). To clarify the role of Src and Syk kinase in inflammatory responses, we additionally determined NO production under Src or Syk kinase inhibitor treatment conditions. As expected, treatment of 4-Amino-3-(4-chlorophenyl)-1-(t-butyl)-1H-pyrazolo[3,4-d]pyrimidine, 4-Amino-5-(4-chlorophenyl)-7-(t-butyl)pyrazolo[3,4-d]pyrimidin (PP2, a Src inhibitor) and piceatannol (a Syk inhibitor) dose-dependently reduced LPS-mediated NO production without altering cell viability (Figure 4e,f). By DAA inhibition on upstream molecules, the transcriptional activity of NF-κB was repressed, resulting in downregulation of inflammatory responses.

### 2.4. The Anti-Inflammatory Effect of DAA in the AP-1 Signaling Pathway

**Figure 5:**
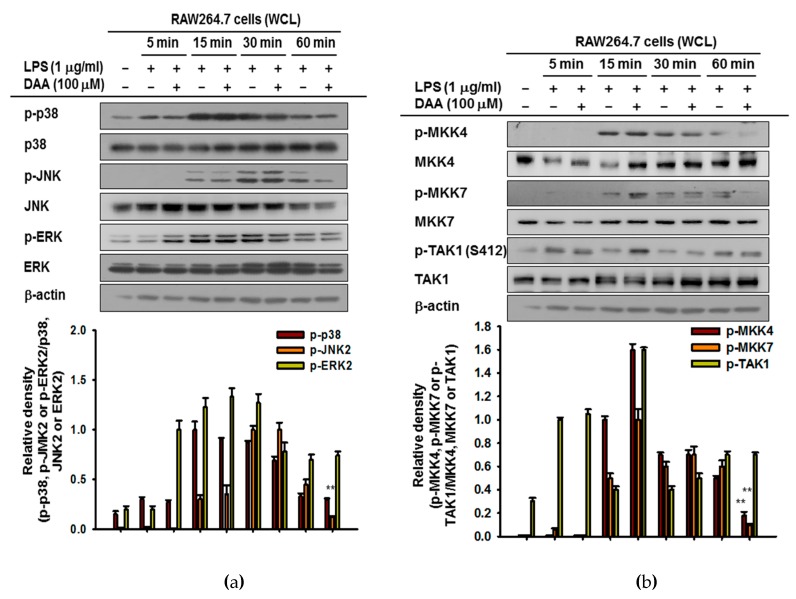

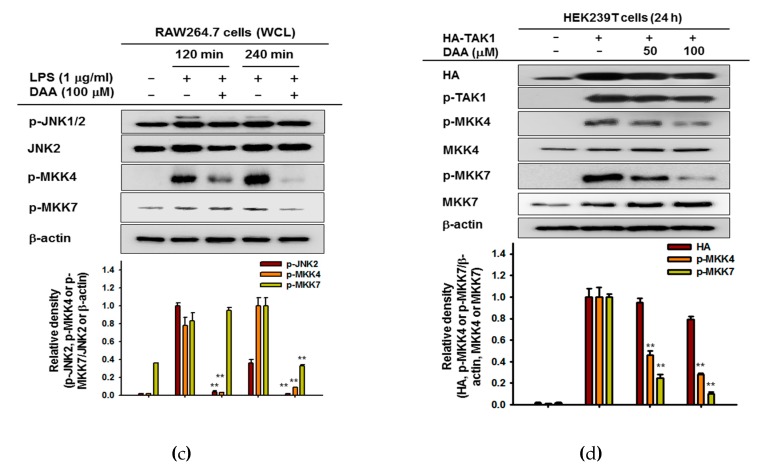
Effect of DAA on the AP-1 cascade. (**a**–**c**) DAA-pre-treated RAW264.7 cells were exposed to LPS (1 µg/mL) time-dependently, and whole-cell lysates were prepared. Phosphorylated or total levels of p38, c-Jun N-terminal kinase (JNK), extracellular signal-regulated kinase (ERK). mitogen-activated protein kinase kinase 4 (MKK4), MKK7, transforming growth factor beta-activated kinase 1 (TAK1), and β-actin were determined by immunoblotting. (**d**) TAK1-transfected HEK293T cells were treated with DAA (0–100 µM) for 24 h. Whole-cell lysates of cells were prepared, and phosphorylated or total forms of TAK1, MKK4, MKK7, HA, and β-actin were detected by immunoblotting. Relative intensity (**b**,**c**) was the calculated ratio using densitometric scanning values of phospho-proteins and densitometric scanning values of β-actin, HA or total forms of proteins from blots observed with independent repeats (*n* = 3) by the DNR Bio-imaging system (Gelquant software Version 2.7). ** *p* < 0.01 compared to the induced group.

Next, we demonstrated the regulatory role of DAA on AP-1 signal cascades. The phosphorylation of MAPKs was ascertained in a time-dependent manner using LPS-exposed RAW264.7 cells with DAA. Among MAPKs, only c-Jun N-terminal kinase (JNK) phosphorylation was downregulated, but phosphorylation levels of p38 and extracellular signal-regulated kinase (ERK) were not affected by DAA (Figure 5a). Then, upstream signaling molecules of JNK were examined. Phosphorylated levels of mitogen-activated protein kinase kinase 4 (MKK4) and MKK7 was reduced by DAA at 60 min (Figure 5b). In addition, the phosphorylation of MKK4 and MKK7 and their downstream protein JNK at 120 and 240 min was found to be reduced by DAA (Figure 5c). However, activated TAK1 (phosphorylated on serine 412) was not regulated by DAA, so we provisionally concluded that the target molecule of DAA in the AP-1 pathway is TAK1. To confirm this, the activation of MKK4 and MKK7 under a TAK1-overexpressed condition was determined. The TAK1-mediated activity of MKK4 and MKK7 were diminished by DAA without altering phospho-TAK1 level (Figure 5d). Using an overexpression strategy, it was shown that DAA repressed inflammatory AP-1 signaling by targeting TAK1.

## 3. Discussion

**Figure 6:**
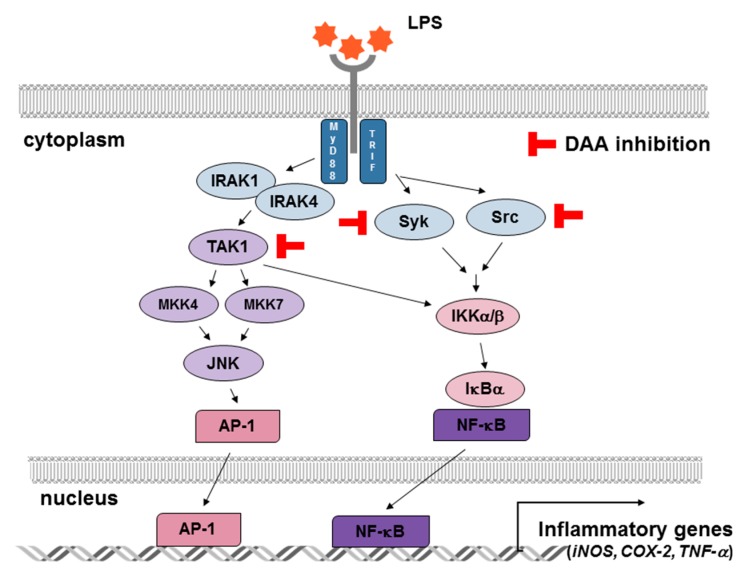
Inhibitory effects of DAA on inflammatory signaling pathways. DAA suppressed the activities of TAK1 in AP-1 cascades and of Syk and Src in NF-κB cascades. These inhibitory actions conclusively reduced the production of inflammatory mediators such as iNOS, COX-2, and TNF-α. T bars are inhibitory action of DAA and black arrows indicate signal transduction.

Dehydroabietic acid is a naturally occurring compound in many coniferous plants [26] and has shown anti-leishmanial, antiaging, and antibacterial activities [19,27,28]. However, the anti-inflammatory mechanism of DAA has not been revealed. In this study, we confirmed that DAA reduced the inflammatory mediator (NO) and inflammatory genes (iNOS and TNF-α) (Figure 2a and Figure 3a). These diminished inflammatory responses were results of TAK1-, Src-, or Syk-inhibiting effects of DAA in AP-1 and NF-κB signaling pathways (Figure 4, Figure 5 and Figure 6).

In adipocytes and macrophages, peroxisome proliferator-activator receptors (PPARs) were activated by DAA, and the secretion of pro-inflammatory cytokines such as monocyte chemoattractant protein-1 (MCP-1), TNF-α, and nitrite was modulated [20,29]. From our data, we screened inflammatory signaling, NF-κB and AP-1, and found target proteins of DAA in inflammatory cascades. Taken together, these findings strongly support that DAA has anti-inflammatory properties with regulation of several proteins. 

Dehydroabietic acid blocked the phosphorylation of Src tyrosine kinase and Syk kinase, a hallmark of activation of these enzyme in NF-κB cascades (Figure 4b,c). Src and Syk kinases in innate cells either initiate or regulate various signaling pathways responding to many stimuli [9]. Activation of Src kinases mediate phosphoinositide 3-kinase (PI3K)/protein kinase B (AKT)/IκBα/NF-κB signaling, and Src kinase plays an essential role to recruit or activate of immune cells [9,24,30,31,32]. The Src family of kinase inhibitors has been considered as anti-inflammatory reagents, for example, dasatinib is used for treatment of chronic myeloid leukemia [33]. Syk kinases are implicated in the initiation of signaling by binding immunoreceptor tyrosine activation (ITAM) domains of Syk and receptors [9,34]. Coupling of Syk and immune cell receptors transduce the signals to regulate cellular responses [35]. Also, Syk kinase could activate not only NF-κB but also MAPKs for the AP-1 pathway [36,37]. Due to these roles, anti-Syk therapeutics for treating inflammatory disorders have been receiving attention, although there is controversial findings in the role of Syk in macrophages. Inhibition of Src and Syk kinases in immune cells suppresses the inflammatory responses, targeting of the pathways mediated by Src or Syk kinases is proposed as a strategy to suppress inflammation [38,39,40]. In these respects, Src- and Syk-targeted DAA has the potential to develop the anti-inflammatory drug.

TAK1 was originally found to be a transforming growth factor (TGF)-β-induced mitogen-activated kinase kinase kinase (MAP3K) in the MAPK pathway. However, it is now known that TAK1 can regulate not only the AP-1 pathway, but also the NF-κB pathway. TAK1 plays a critical role in inflammatory responses by controlling cytokine production including that of TNF-α and IL-8 [41,42,43]. Activated TAK1 induced by TLR ligands could phosphorylate both MAPKs and IKKs [38]. This implies that suppressing TAK1 activity leads to the downregulation of NF-κB and AP-1. In our results, DAA blocked the activation of overexpressed TAK1, so it is possible that NF-κB and AP-1 inactivation results from TAK1 blockade. 

Dehydroabietic acid is a diterpene resin acid that has been traditionally used as herbal medicine [29,41]. Dehydroabietic acid has been reported to exhibit biological activity including antibacterial, antifungal, and anticancer effects [44]. DAA derivatives were already synthesized, and their biological activities evaluated to improve the effect of DAA [44,45]. However, the molecular mechanism underlying the anti-inflammatory effects of DAA is not understood, though DAA plays diverse bioactive roles. We evaluated the molecular mechanism by establishing putative target pathways of DAA linked to TAK1, Src, and Syk, as summarized in Figure 6. In conclusion, DAA is a valuable and natural compound with anti-inflammatory effects. Our findings suggest that DAA could be used as a medicine or cosmetic supplement to ameliorate inflammation.

## 4. Materials and Methods

### 4.1. Materials

Dehydroabietic acid (DAA) was purchased from Ramidus AB (Lund, Sweden). The RAW264.7 cells, a BALB/c-derived murine macrophage cell line (No. TIB-71), and HEK293T cells, a human embryonic kidney cell line (No. CRL-3216), were acquired from ATCC (Rockville, MD, USA). Lipopolysaccharide (LPS, Escherichia coli 0111:B4), polyethylenimine (PEI), PP2, and piceatannol were obtained from Sigma Chemical Co. (St. Louis, MO, USA). 3-(4,5-Dimethylthiazol-2-yl)-2,5-diphenyltetrazolium bromide (MTT) was purchased from Amresco (Solon, OH, USA). Fetal bovine serum (FBS) was from Biotechnics Research (Lake Forest, CA, USA), and RPMI1640 and Dulbecco’s Modified Eagle Medium (DMEM) were obtained from Hyclone (Grand Island, NY, USA). Total and phosphorylated form antibodies against IκBα (Ser32/36), IKKα/β (Ser176/180), Syk (Tyr525/526), Src (Tyr416), ERK (Thr202/Tyr204), JNK (Thr183/Tyr185), p38 (Thr180/Tyr192), MKK4 (Thr261), MKK7g (Ser271/Thr275), TAK1 (Ser412), HA, Myc, and β-actin were purchased from Cell Signaling (Beverly, MA, USA). 

### 4.2. Cell Culture

RAW264.7 cells were cultured in RPMI1640 medium with 10% heat-inactivated FBS and 1% penicillin–streptomycin. The HEK293T cells were incubated in DMEM supplemented with 5% heat-inactivated FBS and 1% penicillin–streptomycin. All cells were housed in a 5% CO_2_ humidified incubator at 37 °C.

### 4.3. NO Production and Griess Assay

The RAW264.7 cells (1 × 10^6^ cells/mL) were plated in a 96-well plate and incubated overnight. Dehydroabietic acid (0–100 µM) was pre-treated for 30 min and incubated in the presence or absence of LPS (1 µg/mL). After 24 h, supernatants of cells were collected, and NO production was determined using Griess reagent as previously reported [25,46]. 

### 4.4. Cell Viability Assay

The RAW264.7 cells (1 × 10^6^ cells/mL) or HEK293T cells (5 × 10^5^ cells/mL) were seeded in a 96-well plate. After preincubation, DAA (0–100 µM) was applied for 24 h. Conventional MTT assay was performed [25].

### 4.5. Preparation of mRNA and Semi-Quantitative PCR

mRNA of LPS-treated RAW264.7 cells was prepared to measure the expression levels of pro-inflammatory molecules. The RAW264.7 cells were pretreated with DAA for 30 min and exposed to LPS for 6 h. Total RNA was isolated with TRIzol reagent following the manufacturer’s instructions. Reverse transcription PCR was conducted [47]. A list of primers used in this study is provided in Table 1.

### 4.6. Plasmid Transfcetion and Luciferase Assay

The HEK293T cells (3 × 10^5^ cells/mL) were seeded in a 12-well plate and incubated overnight. Myc-Syk, HA-Src, or HA-TAK1 plasmids were transfected using PEI for 24 h [48]. Then, cells were treated with DAA and incubated for an additional 24 h. For luciferase assay, HEK293T cells were plated in a 24-well plate. The Flag-MyD88, NF-κB-Luc or AP-1-Luc constructs, and β-galactosidase (as a control) were co-transfected into HEK293T cells using PEI. After 24 h, DAA was additionally applied in a dose-dependent manner for 24 h. Promoter activity assay was performed following Promega’s Luciferase Assay System (Promega, Fitchburg, WI, USA), as previously reported [49]. 

### 4.7. Preparation of Cell Lysates and Immunoblotting Analysis

Cells were washed with PBS once and collected. Cells were centrifuged at 12,000 rpm for 5 min at 4 °C. Cells were lysed with lysis buffer (20 mM Tris-HCl, pH 7.4; 2 mM ethylenediaminetetraacetic acid (EDTA); 2 mM ethyleneglycotetraacetic acid (EGTA); 1 mM DTT; 50 mM β-glycerol phosphate; 0.1 mM sodium vanadate; 1.6 mM pervanadate; 1% Triton X-100; 10% glycerol; 10 µg/mL aprotinin; 10 µg/mL pepstatin; 1 µM benzamide; and 2 µM PMSF). Protein lysates was pelleted by centrifugation (12,000 rpm, 5 min, 4 °C). Supernatant was used for Western blot analysis. The phosphorylated or total forms of IκBα, IKKα/β, Syk, Src ERK, JNK, p38, MKK7, TAK1, HA, Myc, and β-actin were used [38]. Densitometric scanning values of each protein from blots, observed with independent repeats (*n* = 3), were obtained using the DNR Bio-imaging system (Gelquant software Version 2.7, Neve Yamin, Israel). Calculation of relative intensity was carried out with following equation. Relative intensity = densitometric scanning value of phosphoprotein or total protein/densitometric scanning value of corresponding total protein or loading control (β-actin or Lamin C). The highest level of densitometric scanning value of total or phospho-protein in a group was set as 1.

### 4.8. Statistical Analysis

The results were analyzed using either ANOVA/Scheffe’s post-hoc test or the Kruskal–Wallis/Mann–Whitney test. A value < 0.05 was considered statistically significant. All statistical tests were performed using the computer program SPSS (SPSS Inc., Chicago, IL, USA).

## Figures and Tables

**Table 1:** Primer sequences used in the RT-PCR analysis.

Name		Sequence (5′ to 3′)
iNOS	F	CCCTTCCGAAGTTTCTGGCAGCAG
	R	GGCTGTCAGAGCCTCGTGGCTTTGG
TNF-α	F	TTGACCTCAGCGCTGAGTTG
	R	CCTGTAGCCCACGTCGTAGC
COX-2	F	CACTACATCCTGACCCACTT
	R	ATGCTCCTGCTTGAGTATGT
GAPDH	F	CACTCACGGCAAATTCAACGGCA
	R	GACTCCACGACATACTCAGCAC

GAPDH, Glyceraldehyde 3-phosphate dehydrogenase.

## Abbreviations

DAA	dehydroabietic acid
iNOS	inducible nitric oxide synthase
COX	cycloocygenase
TNF	tumor necrosis factor
PAMPs	pattern-associated molecular patterns
PRRs	pattern-recognition receptors
TLRs	toll-like receptors
MyD88	myeloid differentiation primary response 88
IKK	IκB kinase
IL	interleukin
MAPKs	mitogen-activated protein kinases
ERK	extracellular signal-regulated kinase
JNK	c-Jun N-terminal kinase
STAT	signal transducer and activator of transcription protein
MEK	MAPK/ERK kinase
MKK	mitogen-activated protein kinase kinase
TAK1	transforming growth factor beta-activated kinase 1
LPS	lipopolysaccharide
poly(I:C)	polyinosinic-polycytidylic acid
PGN	peptidoglycan
NO	nitric oxide
L-NAME	NG-nitro-L-arginine methyl ester
PEI	polyethylenimine
MTT	3-(4,5-dimethylthiazol-2-yl)-2,5-diphenyltetrazolium bromide
FBS	fetal bovine serum
EDTA	ethylenediaminetetraacetic acid
EGTA	ethyleneglycotetraacetic acid
